# *NpWRKY38* Negatively Regulates Drought Tolerance by Impairing ABA/SA Signaling as Well as Photosynthesis in *Narenga porphyrocoma*

**DOI:** 10.3390/plants15182759

**Published:** 2026-09-09

**Authors:** Shuangcai Li, Haibi Li, Kai Zhu, Rongrong Wang, Yanhang Tang, Hui Zhou, Jinju Wei, Yiyun Gui, Xihui Liu

**Affiliations:** 1 Guangxi Key Laboratory of Sugarcane Genetic Improvement, Key Laboratory of Biotechnological and Genetic Improvement of Sugarcane in Guangxi, Ministry of Agriculture, Guangxi Academy of Agricultural Sciences, Nanning 530007, China; snilsc@126.com (S.L.);; 2 Guangxi South Subtropical Agricultural Science Research Institute, Guangxi Academy of Agricultural Sciences, Longzhou 532415, China

**Keywords:** *Narenga porphyrocoma*, *NpWRKY38*, drought tolerance, ABA signaling, SA signaling

## Abstract

*Narenga porphyrocoma*, a wild relative of sugarcane with high drought tolerance, is a valuable germplasm resource for mining drought-tolerance genes. Weighted gene co-expression network analysis (WGCNA) identified *NpWRKY38*, a WRKY transcription factor that was significantly down-regulated under drought stress, a response that was further recapitulated by exogenous abscisic acid (ABA) treatment and consistent with the presence of ABA-responsive elements in its promoter. *NpWRKY38* overexpression in rice resulted in a significant increase in endogenous ABA content, yet led to insufficient stomatal closure, an attenuated response to exogenous ABA, and a significant reduction in drought tolerance. Transcriptomic analysis revealed that stress-related signaling pathways (e.g., MAPK signaling and plant hormone signal transduction) were activated in the OE lines, whereas photosynthesis, carbon fixation, and chlorophyll biosynthesis pathways were globally suppressed. Specifically, the ABA biosynthetic gene *NCED* was up-regulated, whereas the positive signaling regulator *ABI5* was down-regulated and the negative regulator *PP2C* was up-regulated, thereby impairing ABA signal transduction. In addition, genes involved in salicylic acid (SA) biosynthesis and signaling (*PAL*, *C4H*, *NPR4*, *WRKY45*) were broadly down-regulated concurrently with reduced antioxidant enzyme activities. Furthermore, *NpWRKY38* overexpression exacerbated drought-induced chloroplast damage, as evidenced by a greater reduction in SPAD values and severe chloroplast ultrastructural abnormalities, including swelling, disorganized thylakoid lamellae, and disrupted grana. These three pathways, namely impaired ABA/SA signaling, suppressed photosynthesis, and chloroplast damage, collectively exacerbated the drought-sensitive phenotype of the OE lines. This study demonstrates that *NpWRKY38* acts as a multi-pathway negative regulatory mechanism by interfering with ABA signal transduction, suppressing the SA pathway, down-regulating photosynthetic genes, and compromising chloroplast integrity, providing a potential target for drought tolerance breeding in sugarcane.

## 1. Introduction

Sugarcane (*Saccharum* spp. hybrids) is one of the most important crops for sugar and bioenergy production worldwide, yet its yield and quality are often severely reduced by abiotic stresses such as drought [1,2]. The complex genetic background of cultivated sugarcane and the limited availability of stress-tolerance genes have greatly impeded breeding for drought resistance [3,4]. *Narenga porphyrocoma*, a wild relative of sugarcane, has retained abundant stress-resistance genes through long-term natural selection [5]. However, the molecular mechanisms underlying its drought tolerance, especially the key transcriptional regulators, remain poorly understood, which limits the use of its favorable genes in sugarcane breeding.

WRKY transcription factors constitute one of the largest transcription factor families in plants and are extensively involved in plant growth and development, hormone signaling, and stress responses through specific binding to the W-box element in the promoters of target genes [6,7]. Importantly, accumulating evidence indicates that WRKY proteins play critical regulatory roles in plant drought stress responses, exhibiting remarkable functional diversity and bidirectional effects [8,9]. For instance, ZmWRKY79 in maize and TaWRKY76 in wheat function as positive regulators of drought tolerance, promoting the accumulation of osmolytes and the scavenging of reactive oxygen species through activation of abscisic acid (ABA) synthesis or downstream stress-responsive gene expression, thereby enhancing plant drought resistance [10,11]. Conversely, some WRKY members have been identified as negative regulators of drought tolerance. For example, the group IId subfamily WRKY transcription factors in Arabidopsis thaliana suppress the transcription of DREB1-type genes by forming a WRKY-OBE complex, which maintains normal growth under well-watered conditions but compromises drought tolerance [12]. OsWRKY12 in rice negatively regulates drought tolerance by suppressing ABA signaling and the expression of secondary cell wall biosynthesis genes. RrWRKY56 in rose reduces antioxidant enzyme activity and weakens drought tolerance through direct suppression of *RrUSP* expression [13,14]. These findings underscore the critical importance of identifying and characterizing WRKY members in a species-specific context and highlight the complexity of the WRKY-mediated drought response network. This knowledge gap not only limits our systematic understanding of the molecular regulatory network underlying drought responses in sugarcane and its wild relatives, but also hinders the development of molecular breeding strategies targeting negative regulators for drought resistance.

In this study, we performed weighted gene co-expression network analysis (WGCNA) on previously published genome and transcriptome data of *N. porphyrocoma* under drought stress [5] and identified a drought-repressed WRKY38 gene, designated *NpWRKY38*, as a candidate negative regulator. We hypothesized that *NpWRKY38* suppresses drought tolerance, potentially through interference with ABA-mediated signaling or stomatal regulation. To test this hypothesis, we generated *NpWRKY38* overexpression lines in rice and conducted integrated transcriptomic, physiological, and biochemical analyses to dissect its regulatory function and underlying mechanism. Elucidating the role of *NpWRKY38* not only expands our understanding of WRKY-mediated drought regulation beyond model plants, but also provides a potential genetic target for improving drought resistance in sugarcane and its close relatives.

## 2. Results

### 2.1. Identification of NpWRKY38 as a Negative Regulatory Transcription Factor in Drought Response

**Figure 1:**
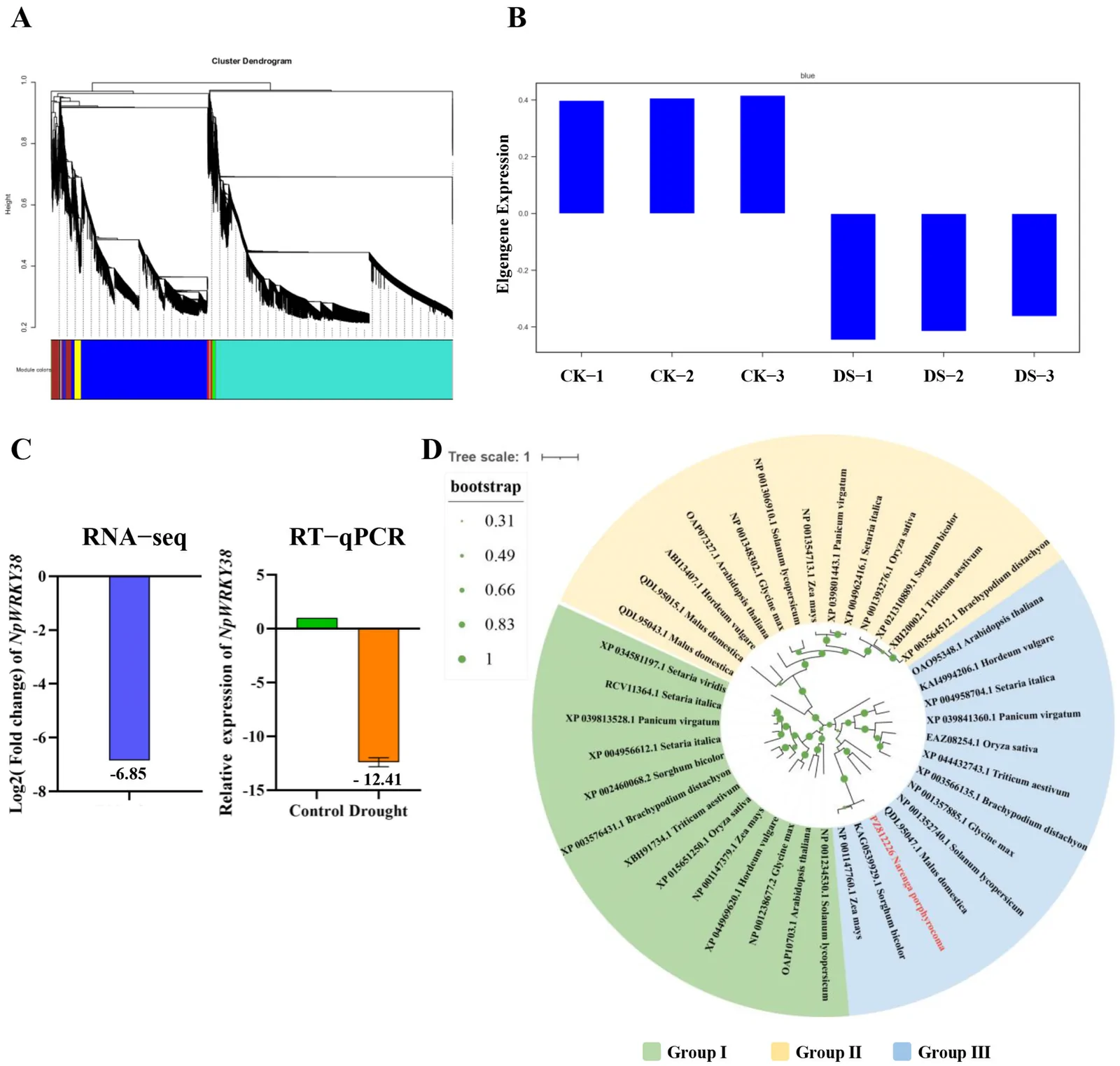
Expression patterns and phylogenetic analysis of the *NpWRKY38* gene. (**A**) Gene cluster dendrogram and module assignment. The dendrogram shows hierarchical clustering of genes based on topological overlap; the colored bar underneath corresponds to different gene co-expression modules. (**B**) Module eigengene expression profile of the blue module across control (CK-1/2/3) and drought-stress (DS-1/2/3) samples. The y-axis represents the module eigengene value. (**C**) Comparison of *NpWRKY38* expression profiles obtained from RNA-seq and RT-qPCR assays. Total RNA was isolated from leaf tissues of Narenga porphyrocoma seedlings. Drought stress was imposed by withholding water for 20 days, and well-watered seedlings were used as the control group. *NpGAPDH* was used as the reference gene for RT-qPCR, and the relative expression level of the control group was normalized to 1. Three independent biological replicates were performed for each group. Log_2_(fold-change) values from RNA-seq and relative expression levels measured by RT-qPCR are presented. (**D**) Circular phylogenetic tree of WRKY proteins from multiple plant species. The tree was constructed using the Maximum Likelihood (ML) method with 1000 bootstrap replicates. Bootstrap support values (≥50%) are shown at each internal node. WRKY members are classified into three subgroups (Group I, Group II, and Group III). NpWRKY38 from *N. porphyrocoma* is highlighted in red. The scale bar represents genetic distance.

To screen for the key transcription factors involved in drought stress responses in *N. porphyrocoma*, we analyzed our previously published genome and drought transcriptome data [5]. WGCNA identified eight distinct co-expression modules, among which the blue module was significantly negatively correlated with drought treatment (DL) (r = −0.29, *p* < 0.05) and positively with the control (CKL) (r = 0.33, *p* < 0.05) (Figure 1A,B), indicating a general down-regulation of its genes under drought. Further functional annotation and differential expression analysis of genes in the blue module identified 535 differentially expressed genes (DEGs), including 87 transcription factors that were significantly down-regulated (Table S2, *p* < 0.05). To find the key candidate genes, we overlapped these DEGs with the top 1% of genes ranked by intramodular connectivity (kWithin) within the blue module. Subsequent functional annotation identified a *WRKY38* transcription factor, which we designated *NpWRKY38*. This gene exhibited the most pronounced decrease in expression under drought treatment, and GO annotation indicated its involvement in the response to salicylic acid (SA) (*p* < 0.01, Figure 1C, Table S3). The drought-repressed expression pattern of *NpWRKY38* was further validated by RT-qPCR using leaf samples from *N. porphyrocoma* seedlings subjected to 20-day water-withholding drought treatment and well-watered controls (Figure 1C). Phylogenetic analysis indicated that NpWRKY38 belongs to Group III of the WRKY family (Figure 1C). Group-III WRKY members are characterized by a single WRKY domain together with a C2HC-type zinc-finger motif, and participate in phytohormone-mediated stress signaling, especially ABA- and SA-dependent abiotic-stress responses, and can act as either positive or negative regulators of drought tolerance [15,16,17,18,19]. This classification prompted us to focus on ABA/SA homeostasis, signal transduction, and downstream transcriptional responses in subsequent functional analyses.

### 2.2. Drought-Induced Down-Regulation of NpWRKY38 Depends on the ABA Signaling Pathway

**Figure 2:**
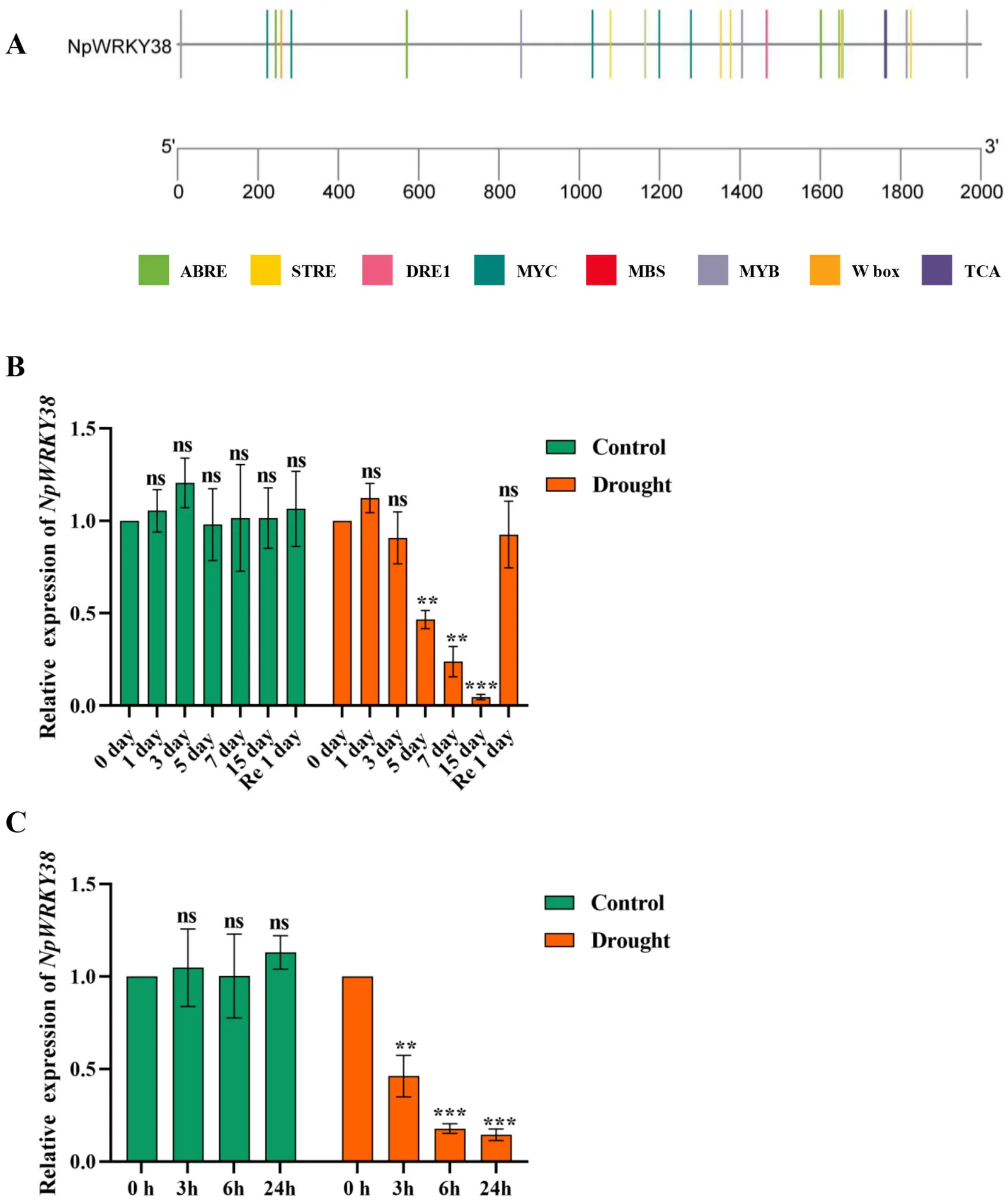
Cis-acting elements in the *NpWRKY38* promoter and its expression responses to drought and ABA treatments. (**A**) Schematic representation of cis-acting regulatory elements in the 2000 bp 5′-upstream promoter region of *NpWRKY38*. Cis-elements were predicted using PlantCARE and visualized with TBtools. Each colored vertical line indicates an individual cis-element, and its position along the horizontal axis corresponds to its nucleotide location on the promoter (0–2000 bp from the 5′-end). (**B**) Relative expression of *NpWRKY38* under drought stress and re-watering treatment. 0 day, 1 day, 5 day, 7 day and 15 day indicate the different durations of drought treatment; Re 1 day represents one day after re-watering. Expression values were normalized to the 0 day control, which was set to 1. (**C**) Time-course relative expression of *NpWRKY38* in response to exogenous ABA treatment. The gene expression level of control sample at 0 h (starting point of treatment) was normalized to 1. 3 h, 6 h and 24 h represent different time points of short-term drought stress, respectively. Green bars indicate well-watered control group; orange bars indicate drought-treated group. Data represent mean ± SD from three biological replicates. Two-way ANOVA followed by Dunnett’s multiple-comparison test was performed. Each group was compared with its corresponding normalized control (0 day for panel (**B**), 0 h for panel (**C**)); ns, no significant difference; **, *p* < 0.01; ***, *p* < 0.001.

To unravel the upstream regulatory signals of *NpWRKY38*, we performed cis-acting element analysis on its 2000 bp promoter region. Numerous cis-motifs associated with phytohormone signaling and stress responses were detected, including ABA-responsive ABRE, salicylic acid-responsive TCA, and jasmonic-acid-related MYC, as well as stress-related STRE, DRE1, MYB, and W-box elements (Figure 2A, Table S4). This suggests that *NpWRKY38* expression may be subject to complex hormonal and transcriptional regulation. To test this hypothesis and characterize its drought-responsive expression dynamics, we examined the transcript levels of *NpWRKY38* in *N. porphyrocoma* leaves at different time points of drought treatment by RT-qPCR. With prolonged drought stress, the expression of *NpWRKY38* began to decrease on day 5, dropping to 0.49, 0.25, and 0.05 times of the control at 5, 7, and 15 days of treatment, respectively (*p* < 0.05, Figure 2B). Rehydration of plants after 15 days of drought restored *NpWRKY38* expression within 1 day (Figure 2B), indicating that its expression is dynamically regulated by water status. Furthermore, treatment of *N. porphyrocoma* seedlings with 50 μM ABA resulted in a significant reduction in *NpWRKY38* expression within 3 h, reaching 0.26-fold of the control at 6 h and remaining at low levels until 24 h (*p* < 0.05, Figure 2C), demonstrating that its drought-induced down-regulation is mediated, at least in part, by the ABA signaling pathway.

### 2.3. NpWRKY38 Overexpression Reduces Drought Tolerance in Rice

**Figure 3:**
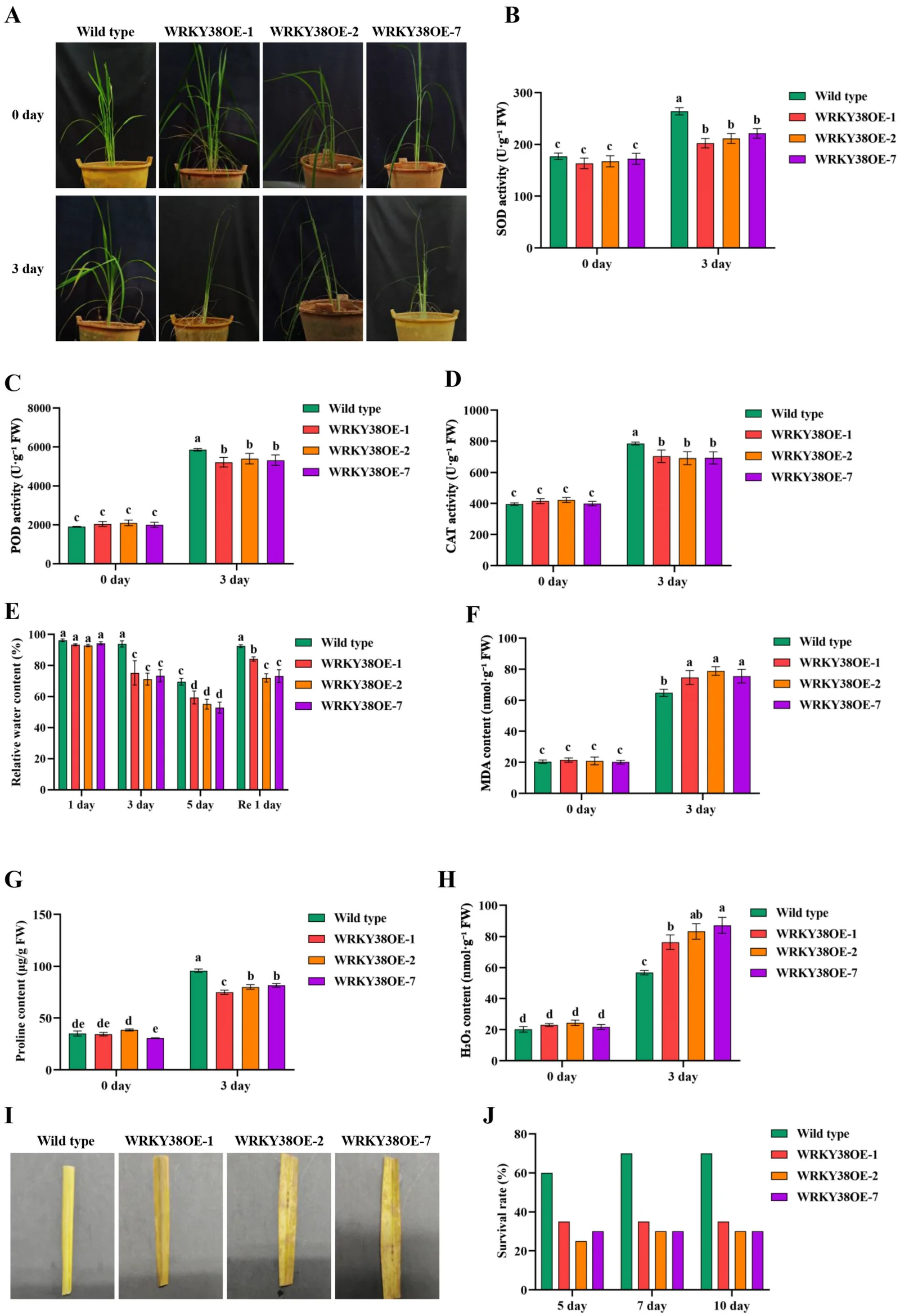
Overexpression of *NpWRKY38* reduces drought tolerance in plants. (**A**) Phenotypic comparison between wild-type and three *NpWRKY38* overexpression lines (WRKY38OE-1, WRKY38OE-2, WRKY38OE-7) under drought stress. 0 day represents well-watered control; 3 day indicates three-day drought-stress treatment. (**B**–**D**) Activities of antioxidant enzymes SOD (**B**), POD (**C**), and CAT (**D**) in wild-type and *NpWRKY38* overexpression plants under normal conditions (0 day) and after 3-day drought treatment. (**E**) Leaf relative water content (%) of wild-type and NpWRKY38 overexpression lines (OE-1, OE-2, OE-7) under 1-, 3-, and 5-day drought stress and subsequent 1-day re-watering. (**F**) MDA content in wild-type and overexpression plants under well-watered condition (0 day) and after 3-day drought stress. (**G**) Proline content of wild-type and overexpression lines at 0 day and 3-day drought treatment. (**H**) H_2_O_2_ content in wild-type and overexpression plants before and after drought stress. (**I**) DAB staining for in situ detection of ROS accumulation in leaves under drought stress. Dark-brown staining indicates ROS accumulation. (**J**) Survival rates of wild-type and *NpWRKY38* overexpression lines at 5, 7, and 10 days after re-watering. Plants were subjected to 10-day drought stress followed by re-watering for recovery. Different lowercase letters represent significant differences at *p* < 0.05 by one-way ANOVA. All data are shown as mean ± SE. For panels (**B**–**J**), each OE line (OE1, OE2, and OE7) was treated as an independent biological replicate, and data from the three lines are shown separately.

To explore the biological function of *NpWRKY38*, we constructed an overexpression vector driven by the CaMV 35S promoter and transformed it into the rice variety Zhonghua 11 (ZH11). A total of 23 PCR-positive transgenic lines were obtained (Figure S1A), from which eight T1 lines with vigorous growth were selected for RT-qPCR analysis, which confirmed that *NpWRKY38* transcript levels were significantly elevated in all overexpression lines compared with wild-type plants (Figure S1B). The three lines exhibiting the highest expression levels, designated WRKY38OE-1, WRKY38OE-2, and WRKY38OE-7, were selected for subsequent drought stress experiments (Figure S1B). Under drought treatment, the overexpression lines exhibited more severe leaf-wilting symptoms than wild-type plants at 3-day drought stress (Figure 3A). Antioxidant enzyme assays revealed that the activities of SOD, POD, and CAT were significantly lower in *NpWRKY38*-OE lines relative to wild-type after 3-day drought stress, with 16.2–23.3%, 7.6–10.8%, and 10.4–11.9% reductions compared with wild-type, respectively (*p* < 0.05; Figure 3B–D). Leaf relative water content (RWC) gradually decreased under progressive drought in both WT and OE lines. However, RWC in all three independent OE lines (OE-1, OE-2, and OE-7) was significantly lower than that of WT from day 1 of drought treatment onward, with reductions ranging from 19.9% to 24.2% at 3-day drought (Figure 3E). After 5 days of drought, the differences further increased, and upon rewatering, the OE lines exhibited markedly impaired recovery compared with WT, with RWC remaining substantially lower than that of WT (Figure 3E). Consistently, *NpWRKY38*-OE lines accumulated higher MDA and H_2_O_2_ contents after 3-day drought stress, which increased by 15.3–21.7% and 34.6–54.0% relative to wild-type (Figure 3F,H). DAB histochemical staining further revealed greater ROS accumulation in the leaves of overexpression plants (Figure 3I). In addition, proline content was markedly lower in *NpWRKY38*-OE lines than in wild-type upon 3-day drought challenge, with a 14.5–21.8% reduction (Figure 3G). After 10-day severe drought stress, plants were rewatered for recovery. The survival rates recorded at 5, 7, and 10 days post-re-watering were substantially reduced in overexpression lines, representing 50.0–57.1% reduction relative to wild-type (Figure 3J). Collectively, these results suggest that overexpression of *NpWRKY38* significantly reduces drought tolerance in rice.

### 2.4. Overexpression of NpWRKY38 Disrupts ABA/SA Homeostasis Under Drought Stress and Thus Impairs Stomatal Closure

**Figure 4:**
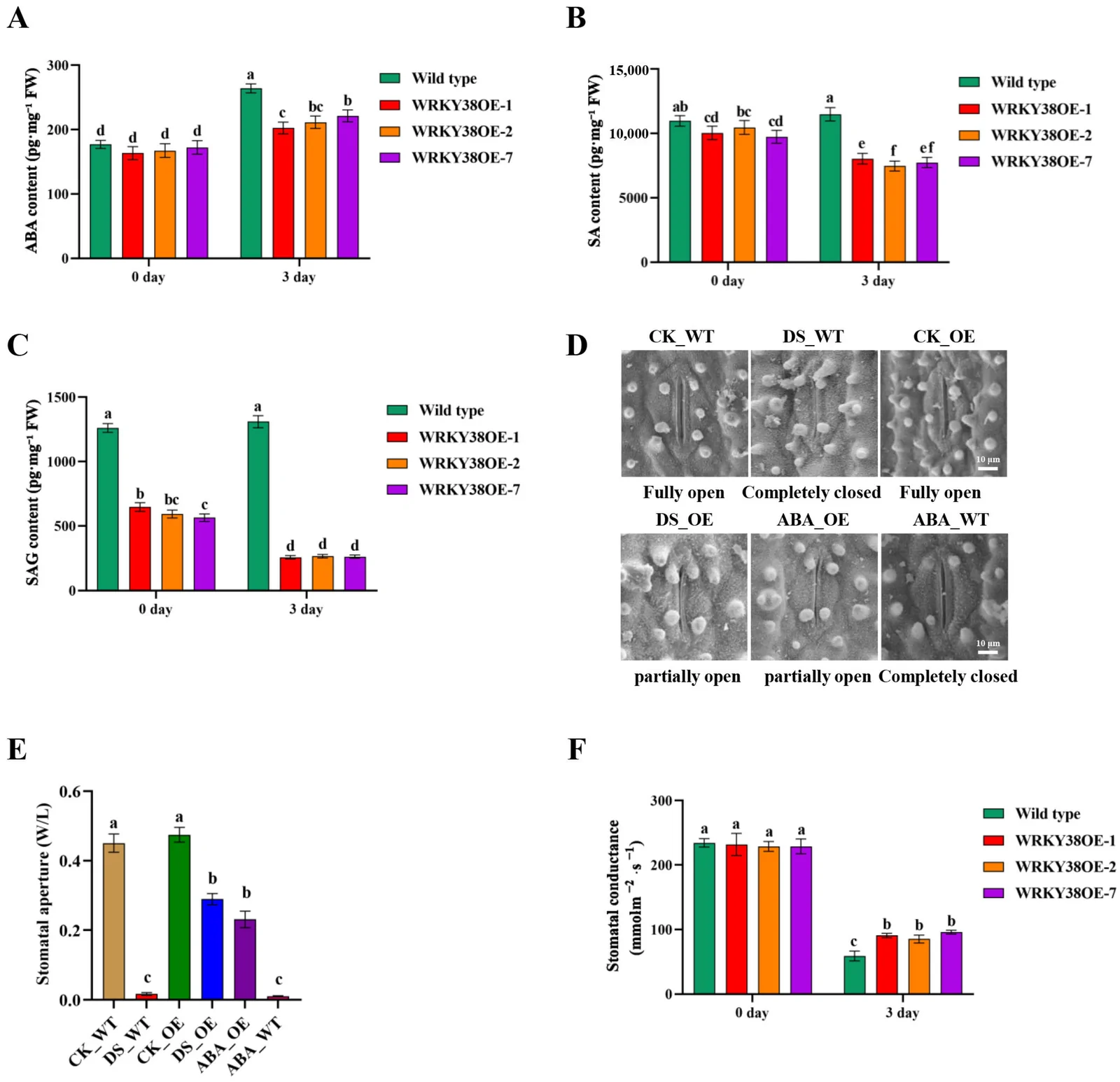
Overexpression of *NpWRKY38* alters ABA and SA contents and impairs stomatal closure under drought stress. (**A**–**C**) Endogenous hormone contents in wild-type (WT) and *NpWRKY38* overexpression plants (WRKY38-OE) under normal condition (0 day) and 3-day drought stress. (**A**) ABA content; (**B**) SA content; (**C**) SAG content. (**D**) Scanning electron microscopy observations of leaf stomata. CK_WT: well-watered wild-type; DS_WT: wild-type under drought stress; CK_OE: well-watered overexpression plant; DS_OE: overexpression plant under drought stress; ABA_OE: overexpression plant treated with exogenous 50 μM ABA; ABA_WT: wild-type treated with exogenous ABA. Scale bar = 10 μm. (**E**) Quantitative measurement of stomatal aperture (width/length, W/L) in different groups. (**F**) Stomatal conductance of wild-type and *NpWRKY38* overexpression lines under drought stress at 0 day and 3 day. Different lowercase letters indicate significant differences based on one-way ANOVA followed by Tukey’s multiple comparison test (*p* < 0.05). For RNA-seq analysis, the three independent OE lines (OE1, OE2, and OE7) were used as three independent biological replicates.

Given that the OE lines exhibited obvious water loss and antioxidant defense reduction under drought stress and that promoter element and GO analyses pointed to potential crosstalk of *NpWRKY38* with ABA and SA signaling, we hypothesized that overexpressing *NpWRKY38* might alter endogenous ABA and/or SA levels. To test this hypothesis, we measured ABA, SA, and SAG contents in leaves of wild-type (WT) and three independent OE lines (WRKY38OE-1, WRKY38OE-2, and WRKY38OE-7) under drought stress. Under well-watered conditions (day 0), no significant differences in basal ABA contents were observed among genotypes. After three days of drought stress, ABA content increased significantly in the WT, and although all three OE lines also showed significant increases, the increases were consistently and significantly lower than that of the WT, reaching only 76.7%, 80.1%, and 83.9% of the WT level, respectively (Figure 4A). In contrast, the levels of both free SA and its inactive storage form, SAG, were significantly lower in all three OE lines under drought stress, with free SA levels decreasing to approximately 65.0–70.0% of the WT levels and SAG levels dropping to about 19.7–20.4% of the WT levels across the three independent lines (Figure 4B,C). This suppression of SA synthesis may be associated with the reduced antioxidant enzyme activities described above.

Despite this significant increase in ABA, the ABA content in OE lines was 1.32-fold higher than that in WT under drought conditions, while the OE lines failed to show any improvement in drought tolerance; their survival rate and RWC remained significantly lower than those in the WT. Given that a primary function of ABA is to induce stomatal closure and thus reduce transpirational water loss, this disconnect between hormone accumulation and physiological response strongly suggests an impairment in ABA signal transduction. To test this hypothesis, we first examined stomatal aperture. Under normal conditions, no significant difference in stomatal aperture was observed between the two genotypes. Under drought stress, the stomata of WT plants nearly completely closed, whereas the OE lines only achieved partial stomatal closure and retained a significantly larger stomatal aperture (Figure 4D,E). Consistent with this observation, measurement of stomatal conductance (Gs) showed that, at day 0 (well-watered), Gs did not differ significantly between the two genotypes. After 3-day drought treatment, Gs decreased substantially in the WT, whereas the OE lines exhibited a significantly smaller decrease, with Gs values in OE-1, OE-2, and OE-7 remaining at approximately 1.54-, 1.45-, and 1.63-fold that of the WT, respectively (Figure 4F). This indicates that the OE lines maintained higher transpiration rates and exhibited severely impaired water retention capacity. These results suggest that either the OE stomata were insensitive to ABA, or that compensatory mechanisms acted upstream of ABA signal transduction. To distinguish between these alternatives, we applied exogenous 50 μM ABA to detached leaves to directly assess stomatal responsiveness to ABA. Stomata of WT leaves exhibited significant closure in response to ABA treatment, whereas those of OE leaves exhibited markedly reduced sensitivity, with stomata remaining partially open and aperture significantly larger than that of WT (Figure 4D,E). This finding confirms an ABA-insensitive phenotype in the OE lines, explaining why substantial endogenous ABA accumulation failed to trigger effective stomatal closure.

### 2.5. Overexpression of NpWRKY38 Suppresses the Photosynthetic Pathway and Disrupts ABA and SA Signaling Pathways

**Figure 5:**
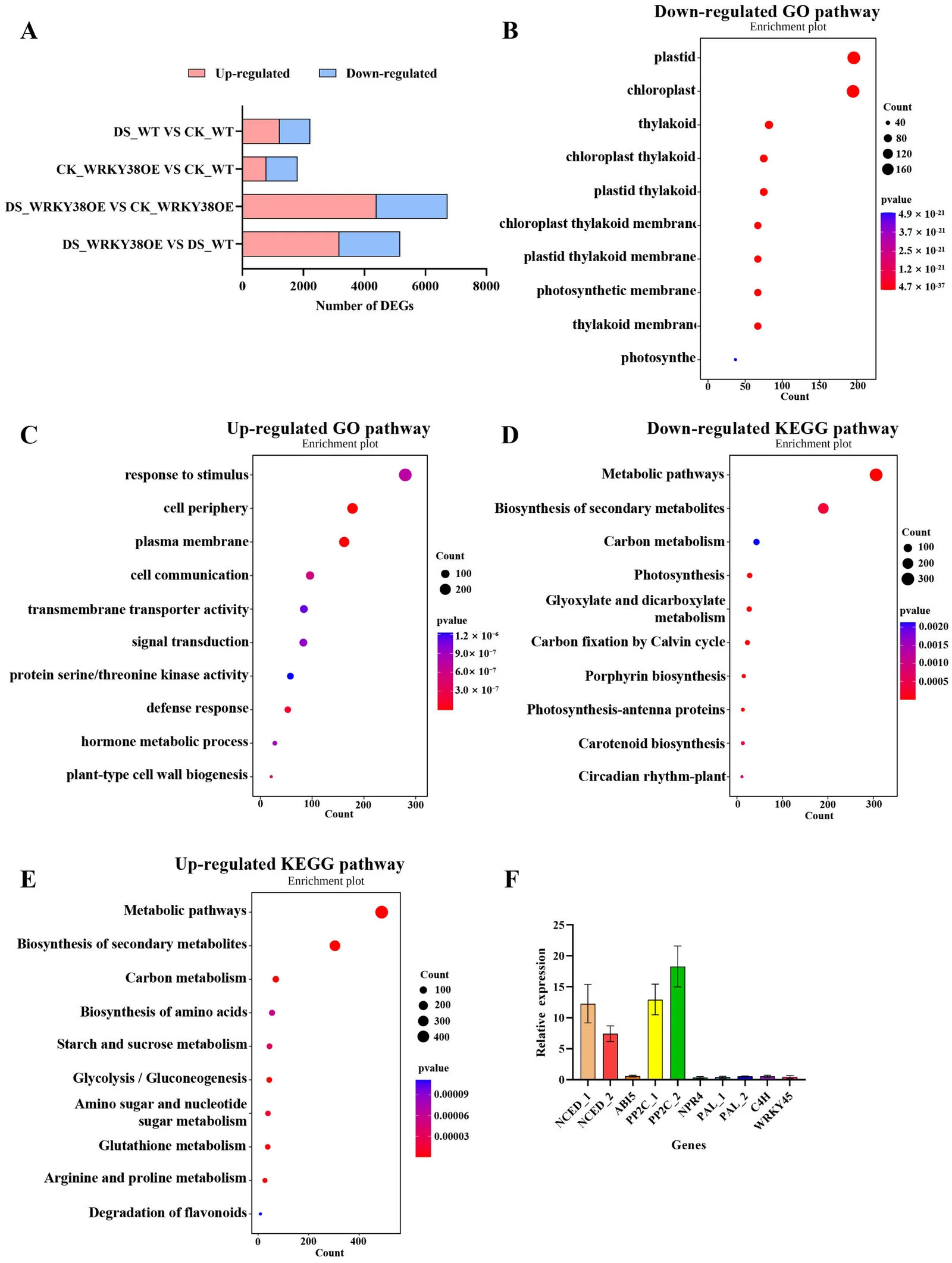
Transcriptomic analysis of *NpWRKY38*-overexpressing (OE) and wild-type (WT) plants under drought stress. For RNA-seq analysis, the three independent OE lines (OE1, OE2, and OE7) were used as three independent biological replicates. (**A**) Number of differentially expressed genes (DEGs) in four comparison groups. Red bars represent up-regulated genes, blue bars represent down-regulated genes. (**B**) GO enrichment analysis for down-regulated *NpWRKY38*-dependent drought-specific DEGs. (**C**) GO enrichment analysis for up-regulated *NpWRKY38*-dependent drought-specific DEGs. (**D**) KEGG enrichment analysis for down-regulated *NpWRKY38*-dependent drought-specific DEGs. (**E**) KEGG enrichment analysis for up-regulated *NpWRKY38*-dependent drought-specific DEGs. (**F**) RT-qPCR validation of selected DEGs from the comparison group DS_WRKY38OE vs. DS_WT. Data are shown as mean ± SD.

To investigate the global transcriptomic changes mediated by *NpWRKY38* under drought stress, we performed RNA-seq analysis using leaf samples from wild-type and three *NpWRKY38*-overexpressing lines (OE-1, OE-2, and OE-7) under control and drought-stress conditions. For RNA-seq analysis, the three OE lines were used as three independent biological replicates. Four pairwise comparisons were conducted to identify differentially expressed genes (DEGs, *p* < 0.05; Figure 5A, Table S5). Under drought conditions (DS_WRKY38OE vs. DS_WT), 3173 genes were significantly up-regulated and 2000 genes were down-regulated in *NpWRKY38*-overexpressing plants (Figure 5A). In the comparison within overexpression lines (DS_WRKY38OE vs. CK_WRKY38OE), drought stress induced 4391 up-regulated and 2333 down-regulated DEGs (Figure 5A). Under normal growth conditions (CK_WRKY38OE vs. CK_WT), relatively fewer DEGs were detected, with 786 up-regulated and 1022 down-regulated genes, indicating that *NpWRKY38* triggers limited transcript-level reprogramming without drought stimulus (Figure 5A). For the wild-type, drought stress caused 1216 genes to be up-regulated and 1004 genes to be repressed (DS_WT vs. CK_WT; Figure 5A). Collectively, these transcriptome data demonstrated that *NpWRKY38* provokes extensive transcript-level reprogramming preferentially under drought conditions, which may contribute to its drought-hypersensitive phenotype.

To further screen the drought-specific downstream target genes regulated by *NpWRKY38*, we eliminated the background DEGs that were independently induced by *NpWRKY38* overexpression under normal conditions or by the intrinsic drought response of rice. The DEGs obtained from the comparison of DS_WRKY38OE vs. CK_WRKY38OE were separately overlapped with the DEGs from CK_WRKY38OE vs. CK_WT and DS_WT vs. CK_WT. In total, 391 DEGs associated with the basal effect of *NpWRKY38* overexpression and 736 DEGs related to the general drought response in wild-type were identified (Figure S2A,B). After removing these two sets of background genes from the DEGs of DS_WRKY38OE vs. CK_WRKY38OE, a total of 5659 DEGs were finally obtained (Table S5). These genes were defined as the core target genes specifically regulated by *NpWRKY38* during rice drought stress, which excluded the interference of genotype background and universal drought response.

To explore the potential biological functions of these 5659 *NpWRKY38*-dependent drought-specific target genes, we performed GO and KEGG enrichment analyses separately for up-regulated and down-regulated genes (Figure 5B,C; Table S6). Among the down-regulated genes, GO analysis revealed significant enrichment in photosynthesis-related terms, including photosynthesis, chloroplast, thylakoid, and plastid-thylakoid membrane cellular components (Figure 5B; Table S6). In contrast, the up-regulated genes were predominantly enriched in stimulus response, defense response, cell periphery, plasma membrane, transmembrane transporter activity, and protein serine/threonine kinase activity (Figure 5B; Table S6). Consistent with GO results, KEGG analysis showed that the down-regulated genes were enriched in photosynthesis, carbon fixation by Calvin cycle, porphyrin biosynthesis, and the carotenoid biosynthesis pathway, whereas the up-regulated genes were enriched in metabolic pathways, biosynthesis of secondary metabolites, carbon metabolism, starch and sucrose metabolism, glutathione-mediated redox metabolism, as well as amino-acid-related metabolic pathways (Figure 5C; Table S6). These enrichment profiles suggest that overexpression of *NpWRKY38* simultaneously activates stress-related signaling pathways while suppressing photosynthetic and energy metabolism under drought. Specifically, the up-regulation of stimulus-response and kinase-associated genes indicates that the OE lines perceive and respond to drought stress at the transcriptional level; however, the concurrent down-regulation of photosynthesis, carbon fixation, and chlorophyll biosynthesis genes severely compromises the plant’s capacity for energy production and carbon assimilation. This dual effect—ineffective stress activation coupled with photosynthetic impairment—likely leads to energy depletion, oxidative damage, and ultimately the enhanced drought-sensitive phenotype observed in the OE lines. This provides a molecular basis for the decreased drought tolerance of *NpWRKY38*-overexpressing lines.

To further characterize the functional consequences of *NpWRKY38* overexpression, we performed GO and KEGG enrichment analyses separately for up-regulated and down-regulated DEGs from the DS_WRKY38OE vs. DS_WT comparison (Figure S2B–E; Table S6). For the down-regulated genes, GO analysis revealed significant enrichment in photosynthesis-related terms, including chloroplast thylakoid membrane, photosynthesis light reaction, and chlorophyll binding (Figure S2B). Consistently, KEGG pathway analysis showed that the down-regulated genes were enriched in photosynthesis, Calvin cycle carbon fixation, secondary metabolite biosynthesis, and circadian rhythm pathways (Figure S2C). In contrast, the up-regulated genes were predominantly enriched in GO terms associated with stress-related signaling pathways, such as cell periphery, plasma membrane, signal transduction, and protein kinase activity (Figure S2D). Correspondingly, KEGG analysis of the up-regulated genes revealed significant enrichment in metabolic pathways, secondary metabolite biosynthesis, plant hormone signal transduction, starch and sucrose metabolism, and MAPK signaling pathways (Figure S2E). Collectively, these results suggest that *NpWRKY38* overexpression simultaneously activates stress-related signaling while suppressing photosynthetic and carbon fixation pathways under drought stress, a dual effect that likely impairs energy production and carbon assimilation, thereby contributing to the reduced drought tolerance of the overexpression lines.

Notably, compared with WT, the OE lines exhibited significant up-regulation of *NCED* genes (*OsJFZ_07G00036600*, *OsJFZ_12G00223800*), which encode key enzymes in ABA biosynthesis. In contrast, the expression of the positive ABA signaling regulator *ABI5* (*OsJFZ_01G00400300*) was down-regulated, while that of the negative regulator *PP2C* was up-regulated (*OsJFZ_01G00236500*, *OsJFZ_12G00069200*, Figure 5F, Table S5). These results indicate a functional impairment in ABA signal transduction. In addition, a broad down-regulation was observed for genes in the SA pathway. These included the key SA biosynthetic genes *PAL* (*OsJFZ_04G00209600*, *OsJFZ_04G00209400*) and *C4H* (*OsJFZ_02G00007000*), together with the central signaling regulator *NPR4* (*OsJFZ_01G00418900*) and its downstream transcription factor *WRKY45* (*OsJFZ_01G00137900*, Figure 5F, Table S5). These transcriptional changes further compromised stomatal regulation and SA-mediated defense responses under drought stress. Collectively, these findings suggest that *NpWRKY38* overexpression results in a global suppression of core photosynthetic genes, leading to reduced carbon assimilation capacity and impaired energy metabolism. This, together with impaired ABA/SA signal transduction, prevents elevated ABA from conferring effective drought protection, ultimately resulting in enhanced drought sensitivity.

### 2.6. NpWRKY38 Overexpression Exacerbates Drought-Induced Chloroplast Damage

**Figure 6:**
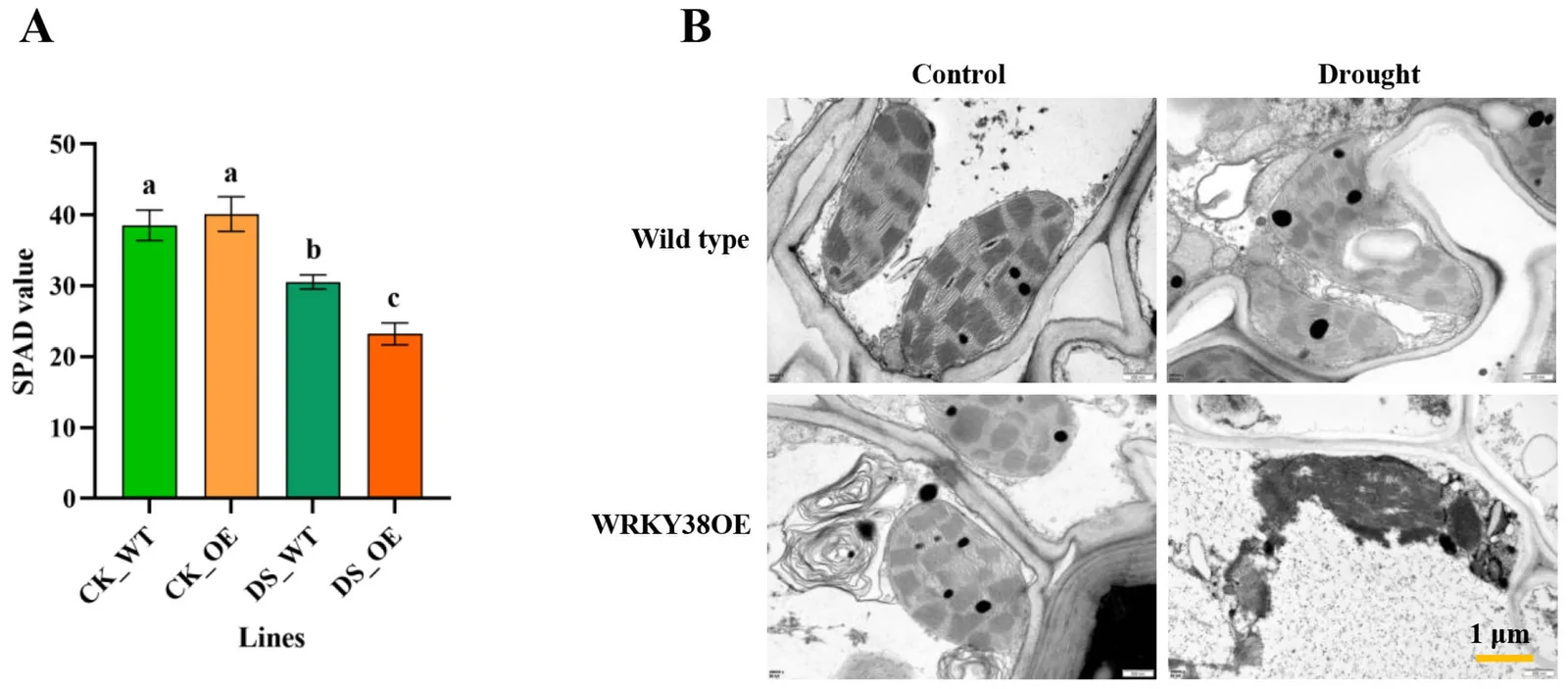
Overexpression of *NpWRKY38* exacerbated drought-induced chlorophyll degradation in rice. (**A**) SPAD values reflecting leaf relative chlorophyll content. CK_WT, well-watered wild-type; CK_OE, well-watered NpWRKY38-OE; DS_WT, wild-type under 3-day drought stress; DS_OE, NpWRKY38-OE under 3-day drought stress. Different lowercase letters indicate significant differences (*p* < 0.05). Data are presented as mean ± SD. (**B**) Transmission electron microscopy observation of chloroplast ultrastructure in leaf mesophyll cells. Scale bar = 1 μm.

Consistent with the notion that down-regulation of photosynthetic genes is often associated with chloroplast damage under drought stress [20,21], our transcriptomic data reveal the significant enrichment of down-regulated genes in the OE lines in photosynthesis-related pathways, particularly those involved in the chloroplast thylakoid membrane, light reactions, and chlorophyll binding. We hypothesized that chloroplast integrity might also be compromised in these plants. To test this hypothesis, we measured the chlorophyll content (SPAD values) and examined chloroplast ultrastructure using TEM. Under well-watered conditions, SPAD values did not differ significantly between the WT and OE lines, and chloroplast ultrastructure was normal, with regularly arranged thylakoids and grana (Figure 6A,B). After drought stress, SPAD values decreased in both lines, but the reduction was significantly greater in the OE lines. The OE lines also exhibited severe chloroplast swelling, disorganized thylakoid lamellae, and disrupted grana (Figure 6A,B). These results indicate that *NpWRKY38* overexpression exacerbates drought-induced chloroplast damage and photosynthetic impairment, thereby contributing to reduced drought tolerance in rice.

## 3. Discussion

Wild relatives of sugarcane harbor abundant drought-tolerance-related genes and serve as genetic resources for drought tolerance breeding [22,23,24]. *N. porphyrocoma*, a wild relative of sugarcane, holds considerable potential for sugarcane breeding [5]. However, its mechanisms of drought tolerance remain unclear. In this study, we identified a WRKY transcription factor, *NpWRKY38*, that was significantly down-regulated under drought stress in *N. porphyrocoma*. Heterologous overexpression in rice confirmed that *NpWRKY38* functions as a negative regulator of drought tolerance. Further analyses revealed that its overexpression impairs stomatal closure by interfering with ABA signaling, compromises antioxidant defense by suppressing the SA pathway, and down-regulates photosynthetic genes, leading to chloroplast structural damage and impaired carbon assimilation. Collectively, these three pathways act synergistically to exacerbate the drought-sensitive phenotype, as evidenced by the greater reductions in relative water content, antioxidant enzyme activities, and survival rates, as well as the more severe chloroplast ultrastructural abnormalities observed in the overexpression lines (Figure 3, Figure 4, Figure 5 and Figure 6).

Extensive crosstalk exists between the ABA signaling pathway and the WRKY transcription factor family [25]. WRKY members can either activate ABA-responsive genes to enhance drought tolerance or suppress ABA signaling to attenuate it [26,27,28]. In this study, *NpWRKY38* expression in *N. porphyrocoma* was down-regulated by drought stress and ABA, and its promoter contained multiple ABA-responsive elements, indicating that *NpWRKY38* functions as a negative regulator downstream of ABA signaling. *NpWRKY38* overexpression in rice resulted in a significant increase in endogenous ABA content under drought stress. However, the OE lines exhibited more severe wilting, lower survival rates, insufficient stomatal closure, and an attenuated response to exogenous ABA. These findings indicate that overexpression did not affect ABA biosynthesis per se, but instead impaired ABA perception or signal transduction. Under drought stress, transcriptomic data comparing the overexpression (OE) lines with wild-type (WT) revealed that *NCED*, a key ABA biosynthetic gene, was up-regulated in the OE lines, consistent with the elevated ABA levels. However, two key components of the ABA signaling pathway exhibited opposite expression patterns: the negative regulator *PP2C* was significantly up-regulated, whereas the positive regulator *ABI5* was significantly down-regulated. During ABA signal transduction, PP2C suppresses downstream signaling by inhibiting OST1 kinase activity, whereas the down-regulation of ABI5, a key transcription factor downstream of OST1, further compromises stomatal closure [29,30,31,32]. Previous studies have shown that some WRKY transcription factors participate in ABA signaling by directly binding to the *ABI5* promoter (e.g., OsWRKY12, AtWRKY40) or by modulating *PP2C* expression (e.g., GhWRKY21, ZmWRKY87) [14,33,34,35]. Therefore, the concurrent up-regulation of *PP2C* and down-regulation of *ABI5* in the OE lines jointly disrupt ABA signaling at both the signal transduction and transcriptional execution levels, providing a mechanistic explanation for the stomatal insensitivity observed despite elevated ABA accumulation.

In addition to impaired ABA signaling, systemic suppression of the SA pathway constitutes another important aspect of the negative regulatory function of *NpWRKY38*. Under drought stress, free SA levels in the OE lines decreased to approximately 67% of those in the WT, and the SA biosynthesis genes PAL and C4H together with the signaling components NPR1 and WRKY45 were all significantly down-regulated, indicating that the SA pathway is broadly suppressed from biosynthesis to signal transduction. Consistently, the activities of antioxidant enzymes SOD, POD, and CAT in the OE lines were significantly lower than those in the WT, whereas the MDA content was significantly higher (Figure 3D–H). Given that SA is known to up-regulate antioxidant enzyme activities and contribute to reactive oxygen species scavenging [36,37,38], the suppression of the SA pathway is likely associated with compromises the antioxidant defense capacity of the OE lines, which is further corroborated by the enhanced DAB staining and elevated H_2_O_2_ levels observed in the OE lines under drought stress (Figure 3H,I). Notably, extensive antagonism exists between ABA and SA signaling [39,40], suggesting that the excessive accumulation of ABA in the OE lines may further inhibit SA synthesis through antagonistic mechanisms, simultaneously impairing both hormone pathways. This reciprocal inhibition likely exacerbates the physiological defects, as the OE lines fail to mount effective stomatal closure and ROS scavenging simultaneously.

In addition to impaired ABA/SA signal transduction and down-regulation of photosynthetic gene expression, overexpression of *NpWRKY38* was exacerbated drought-induced chloroplast structural damage. Under well-watered conditions, SPAD values and chloroplast ultrastructure did not differ significantly between WT and OE lines (Figure 6A,B), indicating that *NpWRKY38* overexpression itself did not directly compromise chloroplast integrity. After drought stress, the OE lines exhibited a significantly greater reduction in SPAD values and displayed chloroplast ultrastructural damage, including chloroplast swelling, disorganized thylakoid lamellae, and disrupted grana (Figure 6A,B). These phenotypes were consistent with the transcriptomic results (Section 2.5), which show widespread down-regulation of photosynthetic genes in the OE lines, particularly those involved in light reaction, chlorophyll binding, and thylakoid membrane organization (Figure 5, Table S6). Previous studies have demonstrated that down-regulation of photosynthetic genes, such as those encoding light-harvesting antenna proteins, is closely associated with chloroplast structural damage [41,42]. For example, LHCB proteins are not only responsible for light capture, but also participate in granal stacking by mediating thylakoid membrane adhesion; their down-regulation can lead to reduced granal width and fewer grana layers [43,44,45]. Therefore, the chloroplast damage observed in the OE lines under drought stress can be at least partially attributed to the global suppression of photosynthetic genes [46,47]. Consistently, our GO and KEGG enrichment analyses of the down-regulated genes specifically reveal significant suppression of photosynthesis-light reaction, carbon fixation, and chlorophyll biosynthesis pathways (Figure 5B,C), providing direct molecular evidence linking the transcriptional repression of photosynthetic machinery to the observed chloroplast ultrastructural defects. Furthermore, suppression of the SA pathway was correlated with reduced antioxidant defense capacity, as evidenced by reduced SOD, POD, and CAT activities (Figure 3D–F), which may have contributed to ROS accumulation and further damage to the chloroplast membrane system [48,49]. Thus, the exacerbated chloroplast injury in OE lines likely results from the combined effects of transcriptional repression of photosynthetic machinery and oxidative damage due to impaired antioxidant defense. Compared with previously reported negative regulators of drought tolerance, *NpWRKY38* exhibits a distinctive multi-pathway suppression strategy. For instance, *OsWRKY12* mainly impairs ABA signaling and secondary cell wall biosynthesis, while *RrWRKY56* directly represses RrUSP to reduce antioxidant capacity [13,14]. In contrast, *NpWRKY38* simultaneously disrupts ABA signal transduction (via up-regulating PP2C and down-regulating ABI5), suppresses the SA pathway (from biosynthesis to signaling), and broadly represses photosynthetic genes. Notably, our transcriptomic analysis revealed that, while photosynthetic and carbon fixation pathways were globally suppressed in the OE lines under drought, stress-related signaling pathways—including MAPK signaling and plant hormone signal transduction—were activated (Figure 5D,E). This dichotomy suggests that the OE lines perceive drought stress and initiate transcriptional responses, but the concurrent suppression of photosynthetic capacity prevents effective stress adaptation, resulting in an energy-deficient state that exacerbates drought sensitivity. This triple impairment explains why OE lines failed to benefit from elevated ABA and exhibited severe chloroplast damage. From a breeding perspective, the strong drought-repressed expression of *NpWRKY38* in *N. porphyrocoma* suggests that its natural function is to prevent over-activation of stress responses under mild drought, thereby balancing growth and survival. In sugarcane, where drought tolerance is a key target, editing the orthologous *NpWRKY38* genes (e.g., via CRISPR-Cas9 knockout or promoter editing to reduce their expression) could unlock enhanced drought tolerance without significant yield penalty. Our transcriptomic data also point to PP2C and ABI5 as potential downstream markers for selecting drought-tolerant germplasm.

Taken together, our results demonstrate that *NpWRKY38* overexpression does not directly target chloroplast structural components; rather, it exacerbates chloroplast damage under drought through the combined effects of down-regulating photosynthetic genes, interfering with ABA-mediated stomatal regulation, and associating with reduced SA-dependent antioxidant capacity. The disruption of chloroplast integrity further compromises carbon assimilation and energy metabolism, ultimately enhancing drought sensitivity in rice [50]. This study thus uncovers a multi-pathway negative regulatory mechanism of a WRKY transcription factor and provides a potential genetic target for improving drought tolerance in sugarcane through gene editing.

## 4. Limitations

Several limitations exist in this study. First, the direct targets of *NpWRKY38* have not been identified by chromatin immunoprecipitation sequencing (ChIP-seq) or DNA affinity purification sequencing (DAP-seq); therefore, whether it directly regulates PP2C, ABI5, or SA pathway genes remains unknown. Second, the lack of a stable genetic transformation system for N. porphyrocoma precludes functional validation of *NpWRKY38* in its native host. Third, the molecular link between ABA overaccumulation and SA suppression in the overexpression lines requires further investigation. Finally, the proposed strategy of editing *NpWRKY38* orthologs for sugarcane breeding awaits experimental validation.

## 5. Materials and Methods

### 5.1. Plant Materials and Treatments

*N. porphyrocoma* plants were obtained from the germplasm resource nursery of our research group and cultivated in a climate chamber under a photoperiod of 14 h light/10 h dark at a temperature of 30 ± 2 °C and a relative humidity of 60–70%. All seedlings were grown in pots (20 cm in diameter and 15 cm in height) filled with field-collected topsoil. Four-leaf-stage seedlings with uniform growth were selected for drought stress treatment. Drought stress was imposed by complete cessation of watering, and no supplementary water was supplied throughout the entire drought period. Leaves were collected at 0, 5, 7, and 15 days after treatment, immediately frozen in liquid nitrogen, and stored at −80 °C for further analysis. For the rehydration treatment, plants were rewatered normally after 15 days of drought, and leaf samples were collected at 1, 3, and 5 days after rewatering. For ABA treatment, seedlings were sprayed with 50 μmol/L ABA solution, and leaves were sampled at 0, 3, 6, 12, and 24 h after treatment. Control plants were sprayed with an equal volume of deionized water containing the same trace amount of ethanol used for ABA dissolution. All treatments were performed with three biological replicates. Rice (*Oryza sativa* L.) variety Zhonghua 11 (ZH11) was used for genetic transformation. Seeds of transgenic and wild-type plants were surface-sterilized with 70% ethanol for 1 min, followed by 2.5% sodium hypochlorite for 20 min, and then rinsed five times with sterile water. The sterilized seeds were sown on 1/2 MS medium. After cultivation at 30 °C under a photoperiod of 16 h light/8 h dark for 7 days, the seedlings were transplanted into pots with field soil and grown in a greenhouse under consistent conditions (16 h light/8 h dark, 30 ± 2 °C, 60–70% relative humidity). For rice drought treatment, watering was completely stopped to naturally reduce soil water content, and the soil moisture was stably maintained at 10–12% throughout the drought treatment period.

### 5.2. Quantitative Real-Time PCR (RT-qPCR)

Total RNA was extracted from leaf tissues of *N. porphyrocoma* and rice using TRIzol reagent. First-strand cDNA was synthesized from 1 µg of DNase-treated total RNA using the PrimeScript™ RT Reagent Kit (Takara, Kusatsu, Shiga, Japan). RT-qPCR was performed using TB Green^®^ Premix Ex Taq™ II (Takara, Kusatsu, Shiga, Japan) on a CFX96 Real-Time PCR System (Bio-Rad, Hercules, Clearwater, FL, USA). The thermal cycling protocol was 95 °C for 30 s, followed by 40 cycles of 95 °C for 5 s and 60 °C for 30 s. Melting curve analysis was conducted to verify amplification specificity. *OsGAPDH* (rice) and *NpGAPDH* (*N. porphyrocoma*) were used as internal reference genes. Relative expression levels were calculated using the 2^−ΔΔCT method. Each reaction was performed with three technical replicates and three biological replicates. Gene-specific primers are listed in Table S1.

### 5.3. Phylogenetic Analysis of NpWRKY38

To determine the evolutionary relationship of NpWRKY38 within the WRKY transcription factor family, a phylogenetic analysis was performed. The full-length protein sequence of NpWRKY38 was retrieved from the *N. porphyrocoma* genome database [5]. Homologous WRKY protein sequences from representative plant species, including *Arabidopsis thaliana*, rice (*Oryza sativa*), and maize (*Zea mays*), were obtained from the NCBI GenBank database (https://www.ncbi.nlm.nih.gov/genbank/ (accessed on 12 March 2025)) using BLASTP (v 2.17.0) searches with the NpWRKY38 protein sequence as the query. Sequences with high similarity and previously reported WRKY proteins from these species were selected for analysis. Multiple sequence alignment was performed using ClustalW (version 2.1) with default parameters. The phylogenetic tree was constructed using the maximum likelihood (ML) method with the best-fit amino acid substitution model and partial deletion of gaps, implemented in MEGA 11 software. Bootstrap analysis with 1000 replicates was performed to evaluate the statistical support for each node. Known Group III WRKY proteins from *A. thaliana* and rice, including AtWRKY70 and OsWRKY12, were included as reference sequences to assist in defining Group III membership.

### 5.4. Promoter Sequence Analysis

The 2000 bp genomic DNA fragment upstream of the start codon of *NpWRKY38* was retrieved from the *N. porphyrocoma* genome database [5] as the promoter region. Putative cis-acting regulatory elements were predicted using PlantCARE (http://bioinformatics.psb.ugent.be/webtools/plantcare/html/ (accessed on 31 May 2025)) [51].

### 5.5. Weighted Gene Co-Expression Network Analysis (WGCNA)

Weighted gene co-expression network analysis was performed using R package WGCNA (version 1.711) to classify genes into co-expression modules [17]. Briefly, a normalized gene expression matrix was used as input, and low-expressed genes were filtered. The soft-threshold power β was selected to satisfy scale-free topology. The adjacency matrix was computed and transformed into topological overlap matrix (TOM). Gene modules were identified by hierarchical clustering and dynamic tree-cutting algorithm via blockwiseModules function with mergeCutHeight = 0.25. Genes belonging to distinct modules were used for subsequent analysis.

### 5.6. Generation of NpWRKY38 Overexpression Rice Lines

Wild-type rice (*Oryza sativa* L. subsp. japonica cv. Zhonghua 11, ZH11) was used in this study. To generate *NpWRKY38* overexpression lines, the full-length coding sequence (CDS) of *NpWRKY38* was amplified from *N. porphyrocoma* cDNA and cloned into the pCAMBIA1301 vector under the control of the CaMV 35S promoter. The recombinant construct was introduced into Agrobacterium tumefaciens strain EHA105 and transformed into mature embryo-derived calli of ZH11 via Agrobacterium-mediated transformation. Transformed calli were selected on medium containing 50 mg/L hygromycin. Resistant calli were transferred to differentiation and rooting media to regenerate T_0_ plants. Genomic DNA was extracted from leaves of putative transgenic plants using the CTAB method, and the presence of the transgene was confirmed by PCR using the gene-specific primer pair NpWRKY38OE-F/NpWRKY38OE-R (Table S1). Wild-type (WT) plants and plasmid DNA served as negative and positive controls, respectively. T_1_ generation lines were obtained by self-pollination of T_0_ positive plants. Three independent homozygous T_1_ lines were selected for subsequent experiments. The expression level of *NpWRKY38* in these lines was examined by RT-qPCR, as described in Section 2.2, with rice *OsGAPDH* as the internal reference gene. All primers used for RT-qPCR are listed in Table S1.

### 5.7. Drought Stress Treatment of Rice

Rice seedlings at the tillering stage were transplanted into plastic pots (20 cm diameter, 15 cm height) filled with homogenized soil, with 20 plants per pot, and grown under a rainproof shelter. For drought treatment, all pots were irrigated to saturation and then water was withheld. Plant phenotypic changes were observed daily at 8:00 and 18:00. Soil water content (SWC) was monitored daily using a portable soil moisture meter (Model ST-S, SanTi Hongke, Weifang, China). When SWC declined to 10–12% (*v*/*v*), it was maintained at this level by daily weighing and replenishing lost water. Control plants were irrigated daily to maintain SWC at 80–90% of field capacity. Each treatment had three biological replicates. After 10 days of drought stress, plants were rewatered to saturation. Survival rates were recorded 5, 7, and 10 days after rewatering.

### 5.8. Quantification of ABA, SA, and SAG (Alicylic Acid-2-O-β-Glucoside) in Rice Leaves

Endogenous ABA, SA, and SAG were quantified with minor modifications to previously reported protocols [52,53]. Briefly, leaf samples were harvested from three independent stable NpWRKY38-overexpressing lines (OE1, OE2, and OE7) and wild-type plants. For each biological replicate, leaf tissues from each OE line were processed separately and analyzed as an independent replicate. Three biological replicates were performed for each line, and the final data are presented as mean ± SD. Then, 50 mg of homogenized leaf powder was extracted using acetonitrile. The obtained extracts were divided into two aliquots. One aliquot was subjected to BPTAB pre-column derivatization at 85 °C for ABA and SA quantification according to Yao et al. (2020) [53]. The second aliquot was directly analyzed without derivatization for SAG measurement following Yang et al. (2024) [52]. Analytes were separated on a Waters HSS T3 column via gradient elution using formic-acid-supplemented water and acetonitrile as mobile phases on a Shimadzu LC-30A UHPLC system (Shimadzu, Kyoto, Japan) and quantified on a Sciex 6500+ triple-quadrupole mass spectrometer (Sciex, Framingham, MA, USA) operating under ESI ± MRM mode. MultiQuant software (v3.0.3) was used for peak integration and standard-curve fitting, with the coefficient of determination R^2^ > 0.99. Derivatized mixed QC standards were injected every 10–15 samples to monitor instrument performance and analytical repeatability.

### 5.9. Scanning Electron Microscopy (SEM) and Transmission Electron Microscopy (TEM)

Leaves were cut into 5 mm × 5 mm segments, fixed in 2.5% glutaraldehyde at 4 °C overnight, dehydrated through a graded ethanol series, and critical-point dried. The samples were then coated with gold using an ion sputter coater. Stomatal morphology on the abaxial leaf surface was observed using a field emission scanning electron microscope (Thermo Fisher Scientific, Eindhoven, The Netherlands, Apreo 2). For stomatal aperture quantification, at least 30 stomata per treatment were randomly selected from ten independent microscopic fields. Stomatal aperture was calculated as the ratio of stomatal width to length (W/L) using ImageJ software (v1.54p, National Institutes of Health, Bethesda, MD, USA). Ten random fields of view were photographed per treatment. Three biological replicates were performed for each treatment.

Leaves were fixed in 2.5% glutaraldehyde, post-fixed in 1% osmium tetroxide, dehydrated through an ethanol series, and embedded in Spurr’s resin. Ultrathin sections (70 nm) were stained with uranyl acetate and lead citrate and examined using a transmission electron microscope.

### 5.10. Measurement of Stomatal Conductance (Gs)

Stomatal conductance was measured between 9:00 and 11:00 a.m. using a portable photosynthesis system (LI-6400XT, LI-COR, Lincoln, NE, USA). The light intensity was set to 1000 μmol·m^−2^·s^−1^, the CO_2_ concentration to 400 μmol·mol^−1^, and the relative humidity to 60–70%. Fully expanded functional leaves were selected from each plant for measurement, and six plants were measured per treatment.

### 5.11. Physiological Measurements

Leaves from wild-type and *NpWRKY38*-overexpressing transgenic rice plants were harvested after 3-day drought treatment. The same batch of leaf materials from wild-type and overexpression lines was used for both physiological measurements and subsequent biochemical assays described below. Relative water content (RWC) was determined by the weighing method previously described [54]. Briefly, the fresh weight (FW) of leaf samples was recorded. Leaf segments were soaked in deionized water until reaching constant weight to obtain turgid weight (TW), followed by oven-drying at 105 °C to constant weight for dry weight (DW). RWC was calculated as RWC (%) = (FW − DW)/(TW − DW) × 100. Three independent biological replicates were set for each genotype under each treatment. For survival rate analysis, seedlings were exposed to 10-day severe drought and then rewatered, and survival rates were recorded at 5, 7, and 10 days after re-watering. Three biological replicates were performed for each genotype.

### 5.12. Biochemical Assays

Using the same set of leaf samples from wild-type and transgenic lines described in Section 5.11. Antioxidant enzyme activities (SOD, POD, CAT), MDA, proline, and endogenous H_2_O_2_ contents were quantified using commercial kits (Suzhou Grace Biotechnology, Suzhou, China). 3,3′-diaminobenzidine (DAB) staining was performed with a plant ROS detection kit (Beijing Solarbio Science & Technology, Beijing, China) to visualize in situ H_2_O_2_ accumulation in leaves. All quantitative measurements were carried out following the manufacturer’s instructions and previously reported methods [55,56]. All biochemical measurements were conducted with three biological replicates for each genotype.

### 5.13. Transcriptome Sequencing Analysis

CK_WT (wild-type, well-watered control), DS_WT (wild-type, drought stress), CK_WRKY38OE (*NpWRKY38*-overexpressing lines, well-watered control), and DS_WRKY38OE (*NpWRKY38*-overexpressing lines, drought stress) were used. For transgenic samples, the three independent OE lines (OE1, OE2, and OE7) were used as three independent biological replicates. Three biological replicates (one from each OE line) were prepared for each group (CK_OE and DS_OE). Total RNA was extracted using the TRIzol method, and RNA integrity (RIN ≥ 7.0) was assessed using an Agilent 2100 Bioanalyzer (Agilent Technologies, Santa Clara, CA, USA). cDNA libraries were constructed using the NEBNext Ultra II RNA Library Prep Kit (NEB, Ipswich, MA, USA) and sequenced on the Illumina NovaSeq 6000 platform (Illumina, San Diego, CA, USA) with a paired-end 150 bp read length. After trimming adapters and low-quality sequences using Trimmomatic (v0.39), the cleaned reads were aligned to the rice reference genome (IRGSP-1.0) using HISAT2 (v2.2.1). Differentially expressed genes were identified using DESeq2 (v1.32.0) with the thresholds |log_2_FC| ≥ 1 and adjusted *p* < 0.05. GO and KEGG enrichment analyses were performed using clusterProfiler (v4.0), with adjusted *p* < 0.05 considered as the significance threshold.

### 5.14. Determination of SPAD Value

Leaf SPAD values were measured using a portable chlorophyll meter (Yaxin-1260, Beijing Yaxinliyi, Beijing, China) on the uppermost fully expanded functional leaf at the middle portion avoiding the main vein between 9:00–11:00 a.m. For each biological replicate, three plants were sampled, and each leaf was measured three times. Three biological replicates were used per transgenic line. For the OE treatment, data from three independent transgenic lines were pooled as a single group.

### 5.15. Statistical Analysis

All experiments were performed with at least three independent biological replicates, and the results are presented as mean ± standard deviation (SD). Statistical analyses were performed using Student’s *t*-test or one-way analysis of variance (ANOVA), followed by Tukey’s HSD test for multiple comparisons. A *p* value < 0.05 was considered statistically significant. Graphs were generated using GraphPad Prism (v9.0) and TBtools (v2.532).

## 6. Conclusions

In summary, we identify *NpWRKY38*, a drought-repressed WRKY transcription factor from the wild sugarcane relative *N. porphyrocoma*, as a negative regulator of drought tolerance. Overexpression of *NpWRKY38* in rice impairs ABA signal transduction (via up-regulation of *PP2C* and down-regulation of *ABI5*), resulting in stomatal insensitivity and defective closure despite ABA accumulation. Concurrently, it suppresses SA biosynthesis, is associated with reduced antioxidant defense, and broadly down-regulates photosynthetic genes, which exacerbates drought-induced chloroplast damage, including swelling, thylakoid disorganization, and grana disruption. These pathways synergistically enhance drought sensitivity in transgenic lines. Our findings reveal a multi-pathway negative regulatory mechanism and provide a potential target for improving drought tolerance in sugarcane through gene editing.

## Data Availability

All relevant data supporting the results of this study are included in the article and its Supplementary Materials. The raw transcriptome sequencing data have been deposited in the Genome Sequence Archive (GSA, https://ngdc.cncb.ac.cn/gsa/ (accessed on 12 March 2026)) under the accession number PRJCA065303. For other reasonable requests, the data can be obtained from the corresponding author or first author. All materials used in this study are available from the corresponding author or the first author upon reasonable request.

## Supplementary Materials

The following supporting information can be downloaded at https://www.mdpi.com/article/10.3390/plants15182759/s1. Table S1. Primers used in this study. Table S2. Differential expression analysis of transcription factors in the blue module. Table S3. Identification of candidate genes in the blue module based on top 1% kWithin. Table S4. Prediction results of cis-acting elements in the NpWRKY38 promoter using the PlantCARE database. Table S5. List of differentially expressed genes. Table S6. GO and KEGG enrichment analysis of commonly down-regulated differentially expressed genes. Figure S1. Molecular identification of *NpWRKY38*-overexpressing *Narenga porphyrocoma* lines. (A) PCR identification of transgenic plants; (B) Relative expression level of *NpWRKY38* in different overexpression lines detected by qRT-PCR, the expression level of wild-type plants was set to 1. Error bars represent standard deviation of biological replicates. Figure S2. Venn diagrams and GO-KEGG enrichment analysis of DEGs in DS_WRKY38OE vs. DS_WT comparison. (A) Venn diagram of DEGs from CK-WRKY38OE_vs_CK_WT and DS-WRKY38OE_vs_CK-WRKY38OE; (B) Venn diagram of DEGs from DS-WT_vs_CK_WT and DS-WRKY38OE_vs_DS_WT; (C) GO enrichment of up-regulated DEGs in DS_WRKY38OE vs. DS_WT; (D) GO enrichment of down-regulated DEGs in DS_WRKY38OE vs. DS_WT; (E) KEGG pathway enrichment of up-regulated DEGs in DS_WRKY38OE vs. DS_WT; (F) KEGG pathway enrichment of down-regulated DEGs in DS_WRKY38OE vs. DS_WT. The size of dots represents gene count, and dot color indi-cates enrichment *p*-value.
